# Comparative Transcriptomic Analysis to Identify the Genes Related to Delayed Gland Morphogenesis in *Gossypium bickii*

**DOI:** 10.3390/genes11050472

**Published:** 2020-04-26

**Authors:** Mushtaque Ali, Hailiang Cheng, Mahtab Soomro, Li Shuyan, Muhammad Bilal Tufail, Mian Faisal Nazir, Xiaoxu Feng, Youping Zhang, Zuo Dongyun, Lv Limin, Qiaolian Wang, Guoli Song

**Affiliations:** 1 State Key Laboratory of Cotton Biology, Institute of Cotton Research, Chinese Academy of Agricultural Sciences, Anyang 455000, China; alimushtaq_caas@yahoo.com (M.A.); pser2010@163.com (H.C.); soomrobabu@yahoo.com (M.S.); lishuyan6688@163.com (L.S.); bilaltufail00@gmail.com (M.B.T.); mfn121@hotmail.com (M.F.N.); bbxe2013@163.com (X.F.); zyp547550790@163.com (Y.Z.); zdy041@163.com (Z.D.); llm0372@126.com (L.L.); zuodongyun@caas.cn (Q.W.); 2 Plant Genetics, Gambloux Agro Bio Tech, University of Liege, 5030 Gambloux, Belgium

**Keywords:** *Gossypium bickii*, RNA-seq, DEGs, WGCNA

## Abstract

Cotton is one of the major industrial crops that supply natural fibers and oil for industries. This study was conducted to understand the mechanism of delayed gland morphogenesis in seeds of *Gossypium bickii*. In this study, we compared glandless seeds of *G. bickii* with glanded seeds of *Gossypium arboreum*. High-throughput sequencing technology was used to explore and classify the expression patterns of gland-related genes in seeds and seedlings of cotton plants. Approximately 131.33 Gigabases of raw data from 12 RNA sequencing samples with three biological replicates were generated. A total of 7196 differentially-expressed genes (DEGs) were identified in all transcriptome data. Among them, 3396 genes were found up-regulated and 3480 genes were down-regulated. Gene Ontology (GO) and Kyoto Encyclopedia of Genes and Genomes (KEGG) annotations were performed to identify different functions between genes unique to glandless imbibed seeds and glanded seedlings. Co-expression network analysis revealed four modules that were identified as highly associated with the development of glandless seeds. Here the hub genes in each module were identified by weighted gene co-expression network analysis (WGCNA). In total, we have selected 13 genes involved in transcription factors, protein and MYB-related functions, that were differentially expressed in transcriptomic data and validated by quantitative reverse-transcription polymerase chain reaction (qRT-PCR). These selected genes may play an important role for delayed gland morphogenesis. Our study provides comprehensive insight into the key genes related to glandless traits of seeds and plants, and can be further exploited by functional and molecular studies.

## 1. Introduction

Cotton is one of the major sources of natural fibers and oil for industries. Despite its industrial importance, the cotton plant is prone to several biotic and abiotic stresses, i.e., environmental stress, insect pests, etc. In response to these stresses, the cotton plant has developed a mechanism of genetic defense, including mechanical protection mediated by the presence of glands on the plant polymers complex that reduce plant digestibility to animals, and toxins that kill or repel herbivores [1]. Besides, the cotton plant has distinct features to attract natural enemies to herbivores [2].

The appearance of pigmented glands on the cotton plant is a distinctive feature of the Malvaceae family [3]. Pigments present on the epidermal tissues of seeds and roots (aerial and cortex) produce terpenoid aldehydes. The presence of gossypol glands reduce the utilization of cotton seed as a source of an edible oil and protein source [4,5]. On the other hand, pigments present on the upper plant surface, i.e., leaves and stem, provide a first line of defense against various insect pests [6,7,8]. Therefore, a cumulative approach to develop cotton cultivars with less glanded seeds (to ensure edibility) and glanded plants (to ensure resistance to insect pests) is a considerable approach for cotton breeders. 

Previous reports have suggested and conferred six genes for gland formation in *Gossypium hirsutum* and *Gossypium barbadense*, like *gl1* that controls gland formation in leaf and cotyledon [9]; gl2gl2gl3gl3 expression of these genes produces glandless character on the whole plant [10,11]. Meanwhile, *gl4* and *gl5* confer a reduction in the number of glands in the cotton plant, and *gl6* controls gland formation in leaf and cotyledon [12,13]. Furthermore, studies have identified multiple alleles controlling gland development and pigment formation in cotton species [14,15].

Understanding the genetic mechanism behind gland formation has opened new dimensions towards producing glandless seeds in cotton, which could provide a revolutionary solution to eliminate hunger and protein shortages for human consumption. Recent advances in genomics and sequencing technology have opened new horizons in understanding the mechanisms underlying complex traits. However, despite all technical breakthroughs, there are few reports suggesting a cotton genome response in delayed gland morphogenesis. The transcriptomic studies remain effective in cotton for identification of differentially-expressed genes which control the gossypol contents of cotton [4,16,17,18]

Rapid advancements in new generation sequencing technologies have shed new light on the research of genetic issues, including those in plant sciences. However, many studies focused on the screening of differentially-expressed genes have associated as much attention to the high degree of interconnection between genes, where genes with similar expression patterns may be functionally related [19,20,21]. *Gl2e* was the first pigment gland-related gene that was identified encoding an MYC transcription factor controlling pigment gland formation and gossypol content in cotton [1,22].

Wild progenitors are an excellent source to identify useful variation in crops. Here we used *Gossypium bickii* (Australian wild species) as a source plant for the identification of differentially-expressed genes in cotton imbibed seeds and seedlings. There are two wild Australian species, *G. bickii* as well as *Gossypium australe,* possessing unique characteristics of having glandless seeds and glanded plants. Recently, a gene related to gland in *G. australe* was published, but the mechanism for delayed gland morphogenesis is still unclear [23]. Here, we used transcriptomic data for imbibed seeds and seedling stages of *G. bickii* to understand and evaluate the genetic mechanism behind gland formation at the seedling stage. In this study, we have identified putative genes for gland formation in *G. bickii*. Furthermore, we have investigated the genetic network involved in gland formation of cotton plants.

High-throughput RNA sequencing data and weighted gene co-expression network analysis (WGCNA) polymerization modules data have been used in this study to sort out the key genes related to the glandless trait in cotton through transcriptomic analysis. Furthermore, quantitative reverse-transcription polymorphic chain reaction (qRT-PCR) technology has been used to validate the expression of selected genes from RNA sequencing (RNA-seq) data.

## 2. Materials and Methods

### 2.1. RNA Extraction, Library Construction and RNA-Seq Analysis

Total RNA was isolated from glandless imbibed seeds of *G. bickii*, glanded imbibed seeds of *Gossypium arboreum*, and glanded seedlings of *G. bickii* and *G. arboreum*. The seeds were treated with water and grown on filter paper in the incubator at a temperature of 30 to 32 °C. After 24 and 48 h, we collected the samples and labeled them as seeds and seedling stages. Total RNA was isolated using the EASY-spin plant RNA kit (Aidlab, Beijing, China), according to the manufacturer’s instructions. cDNA was synthesized using a Prime Script^TM^ II 1st strand cDNA synthesis kit (Takara, Dalian, China) according to manufacturer’s instructions. We used a total of four different samples with three biological repeat methods, 12 RNA-seq libraries and sequenced. A total of 3 µg of RNA from each sample was used to construct the transcriptomic libraries. RNA degradation and contamination were checked using 1% agarose electrophoresis gel, and RNA purity was checked with the NanoPhotometer^®^ spectrophotometer (Implen, Health Care Facilities & Svcs, La Baya Drive West Lake, CA, USA). The RNA sequencing process was accomplished by Novogene Technologies Corporation, Ltd. (Beijing, China) using Illumina HiSeq™ 2000.

### 2.2. Analysis of Differentially-Expressed Genes

To identify differentially-expressed genes (DEGs) in glandless imbibed seeds and glanded seedlings, we used the edgeR package (v1.18.0) in 12 RNA-seq libraries. The data generated through edgeR were used in the express program to generate raw read counts. The DEGs were identified with a fragment per kb per million of the mapped reads (FPKM) value of >0.5, and a false discovery rate (FDR) of <0.01. The FDR was used to determine the *p*-value of 0.005, and a log2-fold change (FC) of 1 was determined as the significant value of differential expression. Sequencing data of 12 RNA-seq have been uploaded to the NCBI database with accession number PRJNA625620. 

### 2.3. Quantification, Gene Ontology and KEGG Pathway in Differential Gene Expression

We estimated the gene expression levels by counting reads of mapped genes, FPKM, with HTSeq v 0.6.1 software to count the number of mapped genes [24]. Demonstrating the number of genes with different expression levels; this was the most common method used for estimating gene expression level [25]. Therefore, we used 4 biological replications, for each sequenced library, and the read counts were adjusted by the edgeR package by one scaling normalized factor. Differential expression analysis of two conditions was performed using the DEG-seq R package v 1.18.0 and DEG-seq. The *p*-value was adjusted using the Benjamini and Hochberg method, followed by the genes being adjusted with a *p*-value of <0.05. Furthermore, gene ontology enrichment analysis of differentially-expressed genes (Kyoto Encyclopedia of Genes and Genomes, KEGG) is a database resource, used to simplify the high-level functions and uses of the biological system (http://www.genome.jp/kegg/). To test the statistical enrichment of KEGG pathways, KOBAS software [26,27] was used.

### 2.4. Construction of Gene Network

The WGCNA analysis was performed to construct a gene co-expression network using R package [28,29]. Co-expression network analyses were conducted to determine the relationship among genes responsible for the delayed gland morphogenesis trait. We took four samples (two samples for *G. bickii* and two for *G. arboreum*) from two different stages (imbibed seeds and seedlings); each sample with three technical and biological repeats as an individual dataset (12 samples) was used for network analysis. To complete the co-expression analysis, the edges file was sorted by weight value, and then 200 pairs of network connections were used to establish an interaction network among genes. The hub genes were screened out on module basis membership (K_ME_) values. The interaction networks between genes were constructed by Cytoscape software 3.7.2 version [30].

### 2.5. qRT-PCR Analysis to Validate RNA-Seq Data

Total RNA was isolated from imbibed seed and seedlings, using the Tiangen RNAprep Pure Plant kit (Tiangen biotech, Beijing, China), according to the manufacturer’s protocol. qRT-PCR was used to confirm the RNA-seq data. Genes related to delayed gland morphogenesis of the transcriptome data were selected and specific primers for RT-PCR were designed using Oligo 7 software. The primers of 13 selected genes were synthesized by Sangon Biotech (Shanghai, China). cDNA was synthesized from RNA and used as a template to make the reaction for qRT-PCR by using Takara qPCR kit SYBR Premix Ex Taq^TM^ II (Tli RNaseH Plus). We have performed each reaction with three biological and three technical replicates on a ThermoFisher Scientific QuantStudio^®^ 5 instrument (Applied Biosynthesis, Foster City, CA, USA). The qPCR circulation conditions included denaturation at 95 °C for 30 s, 45 cycles at 94 °C for 5 s, and annealing and extension at 60 °C for 30 s. The relative expression was calculated for each sample by using the 2^−△△Ct^ method [31].

## 3. Results

### 3.1. Summary of Transcriptome Data

**Figure 1:**
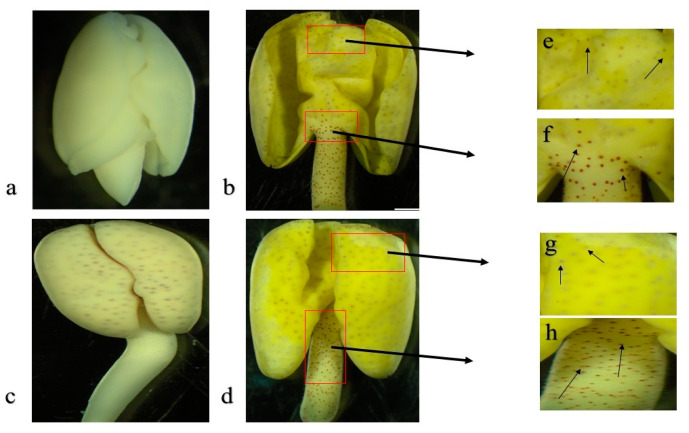
Pictorial description of delayed gland morphogenesis in *Gossypium bickii* and regular gland formation in *Gossypium arboreum* showing glands on seeds and germination stages. (**a**) Imbibed seed image of *G. bickii* showing no glands; (**b**) seed germination stage of *G. bickii* showing glands on cotyledons and hypocotyl; (**c**,**d**) image of *G. arboreum* imbibed seed and germination showing glands; (**e**,**f**) gland formation in cotyledon and hypocotyl of *G. bickii*; and (**g**,**h**) gland formation in cotyledon and hypocotyl of *G. arboreum*.

*Gosssypium bickii* possesses special characteristics, such as having a glandless seed which transforms to glanded at germination stages (Figure 1). Similarly, in lateral plant growth stages including stem, leaves and flowers, glands can be observed. Thus, *G. bickii* could be an excellent source to understand the molecular mechanism of genes related to delayed gland morphogenesis, which controls/regulates gland formation in cotton. This experiment consisted of 12 RNA-seq libraries from imbibed seeds and germination stages of *G. bickii* and *G. arboreum* with three biological replications. A total of 975.52 million raw reads were obtained, and filtered for low quality reads, resulting in 746.22 million clean reads (approximately 131.33 Gb raw data) with an average of 10.94 Gb for each sample. Over 92.17% of the (Q30) values and not less than 43.04% GC contents were observed from the RNA-seq results. The average (Q30) value was 92.93% and GC contents were 43.47%. The clean reads were mapped to the reference genome of *G. arboreum* using TopHat2 software. A total of 96.42% of the clean data was successfully matched to the reference genome, of which 93.11% and 3.31% constituted unique and multiple reads, respectively (Table 1). The above stated results implied the reliability of our transcriptomic data.

**Figure 2:**
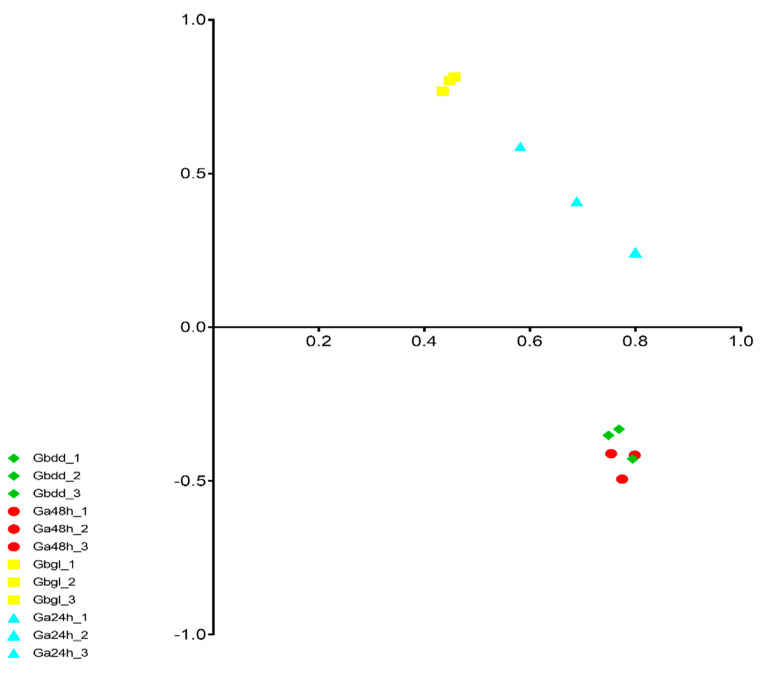
Principal component analysis (PCA) of genes identified from 12 samples with three biological replicates. Gbdd, Ga24h, Gbgl and Ga48h represent glanded seedlings of *G. bickii*, glanded imbibed seeds of *G. arboreum*, glandless imbibed seeds of *G. bickii* and glanded seedlings of *G. arboretum*, respectively.

To further exploit RNA-seq results, we employed principal component analysis (PCA). PCA was performed using RNA-seq data of four samples with three biological replications. This analysis differentiated the glanded and glandless types into different groups. Gbgl samples showed a high degree of differentiation from other samples, while Gbdd and Ga48h were clustered together. Our results for PCA analysis confirmed the differential behavior of glandless *G. bickii* (Figure 2).

### 3.2. Transcriptome Changes during Imbibed Seed (Glandless) and Seedling (Glanded Stages)

**Figure 3:**
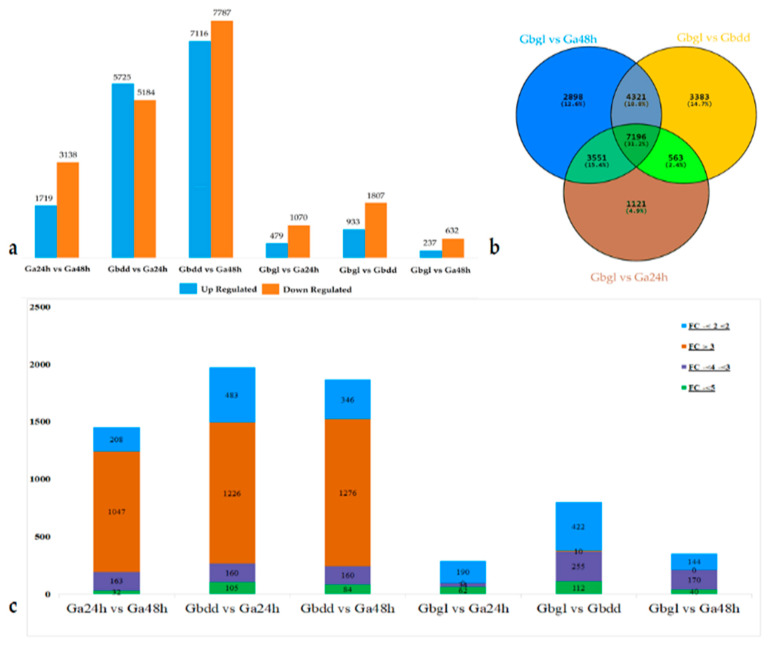
Expression dynamics changes and comparative analysis of differentially-expressed genes (DEGs) between Gbgl, Ga24h, Gbdd and Ga48h following delayed gland morphogenesis at imbibed seed and seedling stages. (**a**) Number of DEGs showing the up-regulated and down-regulated genes; (**b**) Venn diagram showing common genes, all differentially-expressed genes in different stages; (**c**) the number of transcripts demonstrating changes in expression in Gbgl, Ga24h, Gbdd and Ga24h following pattern fold change (FC) in expression, calculated as the log2 ratio of gene expression in glandless imbibed seed with glanded imbibed seed and seedling. Gbdd, Ga24h, Gbgl and Ga48h represent *G. bickii* glanded seedlings, *G. arboreum* glanded imbibed seeds, *G. bickii* glandless imbibed seeds and *G. arboreum* glanded seedlings, respectively.

To explore the DEGs related to delayed gland morphogenesis in different stages viz. imbibed seed and germination, FPKM of the mapped reads values were employed to measure gene expression quantity, followed by the generalized fold change (GFOLD) algorithm to identify DEGs among Gbgl, Ga24h, Gbdd and Ga48h. The total number of genes exhibiting either up-regulation or down-regulation in the respective samples were compared to each other. We identified 35,827 genes between multiple samples. The samples Gbdd vs. Ga48h showed highest number of 14,903 DEGs; among them, 7116 were up-regulated and 7787 were down-regulated. In the samples of Ga24h vs. Ga48h, there was a total of 4857 genes; there were 3138 down-regulated and 1719 up-regulated genes. In samples Gbdd vs. Ga24h, there were 10,909 genes identified, including 5184 down-regulated and 5725 up-regulated-genes. Among Gbgl vs. Ga24h, a total of 1549 DEGs were identified, 1070 down-regulated and 479 up-regulated genes. In Gbgl vs. Gbdd, there was a total of 2740 DEGs identified, 1807 down-regulated and 933 up-regulated genes. The minimum number of DEGs, 869, were identified in Gbgl vs. Ga48h; 237 genes were up-regulated and 632 genes down-regulated (Figure 3a).

**Figure 4:**
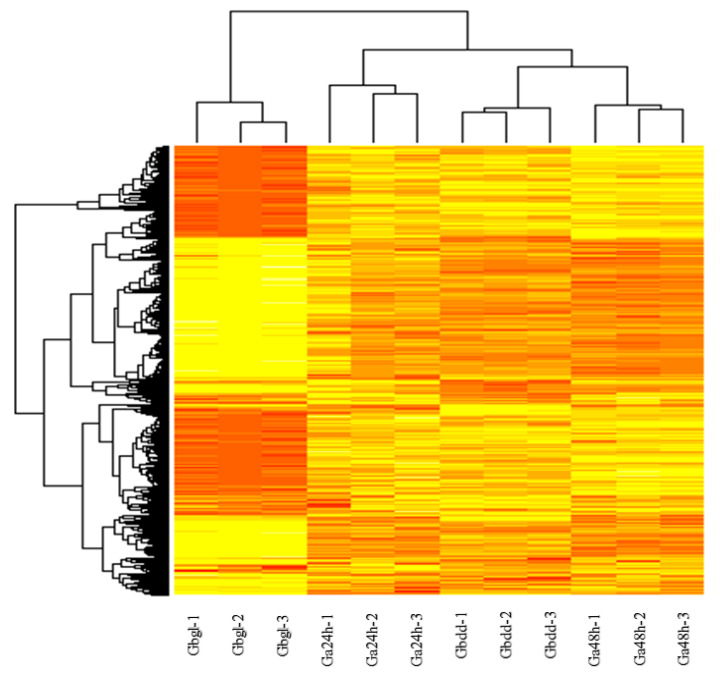
The expression pattern of DEGs from different biological replicates. A heat map represents the relative expression levels of genes based on fragment per kb per million of the mapped reads (FPKM) values using RNA sequencing (RNA-seq) data. Gbdd, Ga24h, Gbgl and Ga48h represent *G. bickii* glanded seedlings, *G. arboreum* glanded imbibed seeds, *G. bickii* glandless imbibed seeds and *G. arboreum* glanded seedlings, respectively.

To further explore the DEGs between different groups, we compared glanded imbibed seeds and seedlings with glandless imbibed seeds of *G. bickii*. We sorted the common genes using a Venn diagram online tool (https://bioinfogp.cnb.csic.es/tools/venny/) as either up-regulated or down-regulated with significant expression level. A total of 7196 DEGs were obtained from whole transcriptomic data, including 3480 down-regulated genes and 3395 up-regulated genes (Figure 3b). The total mapped reads of all differentially-expressed genes were done using DEG-seq (*p*-value <0.01) with expression of gene and the level calculated as FPKM. Further analysis of the log2 ratio found that the percentage of DEGs was mainly distributed as FC >3 and from −2 to 2, -<3 to -<4 and -<5; the percentage of DEGs was distributed widely (Figure 3c). In DEGs among different transcriptomic libraries of Ga24h vs. Ga48h, Gbdd vs. Ga24h and Gbdd vs. Ga48h, the maximum number of genes were identified in FC >3, while the minimum number of genes were identified in FC -<5. In Gbgl vs. Ga24h, Gbgl vs. Gbdd and Gbgl vs. Ga48h, the maximum number of genes were sorted in FC -<2 to <2, and we did not find a single gene in samples Gbgl vs. Ga24h and Gbgl vs. Ga48h under FC >3. In the clustering analysis of FPKM values of DEGs, four samples with three biological repeats were carried out, after integrating the sequencing data from the libraries; the expression of DEGs is shown in the heat map (Figure 4).

### 3.3. Functional Annotations of Differentially-Expressed Genes

**Figure 5:**
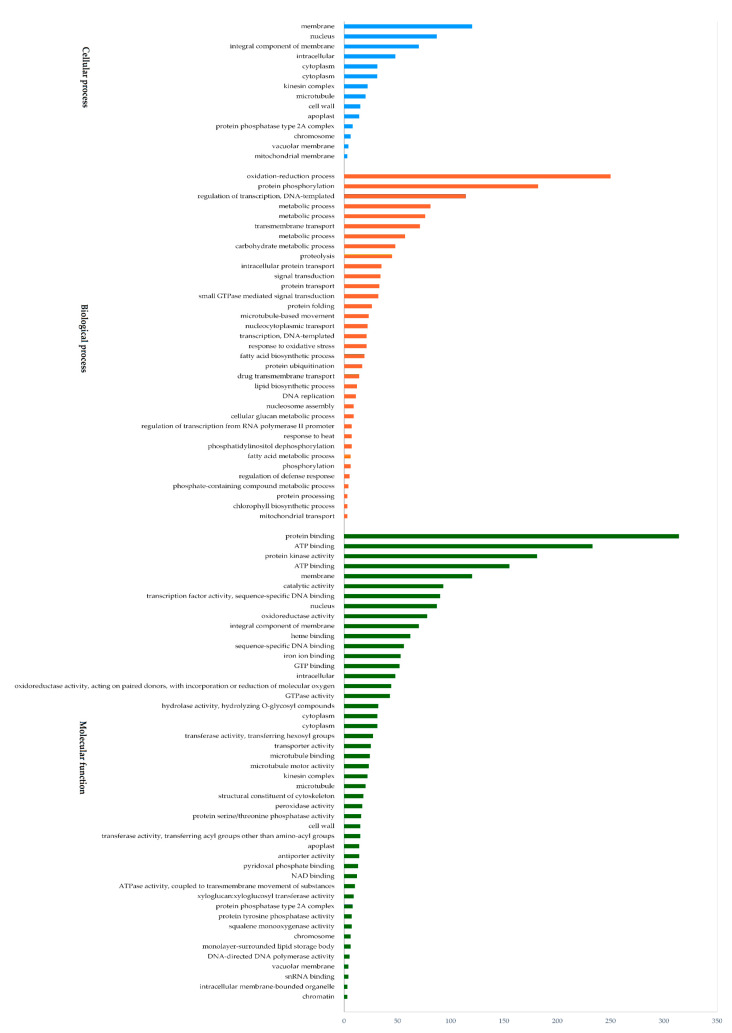
Gene ontology functional classification of DEGs.

With the aim to understand the molecular mechanism of delayed gland morphogenesis and their related genes, the identified 7196 DEGs were classified into 148 Gene Ontology (GO) annotations, including biological processes, cellular components and molecular functions. In molecular function, there were 56 different groups identified; 314 (4.36%) DEGs were involved in protein binding, followed by 233 (3.23%) DEGs in ATP binding and 181 (2.51%) DEGs found in protein activity. In the cellular component category, there were 17 different groups identified with the maximum numbers; 120 (1.66%) DEGs were involved in membrane, 87 (1.20%) DEGs in the nucleus and 70 (0.97%) DEGs in the integral component of the membrane. Then, in the biological processes category, there were 56 subcategories; 250 DEG (3.47%) genes were found in the oxidation–reduction process, followed by 182 (2.52%) DEGs in protein phosphorylation, 157 (2.18%) DEGs in the metabolic process, and 114 (1.58%) DEGs were identified in regulation of transcription, DNA-templated functions (Figure 5 and Table S1). Furthermore, we explored the DEGs in up-regulated and down-regulated genes; GO enrichment and KEGG pathway analysis were performed using Blast2GO software. Here we describe GO of up-regulated and down-regulated genes separately. In total, 3396 up-regulated genes were annotated and categorized into seven GO terms based on molecular function and biological processes except for the cellular component. Under the molecular functions, most up-regulated genes were involved in protein binding, (196 genes, 5.77% of the total up-regulated 3396 genes) and only eight (0.83%) genes were identified in ubiquitinyl hydrolase activity. In biological processes, the maximum number of 21 (0.61%) genes were found in transcription, followed by 19 (0.55%) genes in protein folding, nine (0.26%) genes in protein deubiquitination, and five (0.14%) genes in response to heat. These were the principal subcategories of GO analysis in which up-regulated genes were identified. Down-regulated genes were categorized and annotated into five GO terms based on biological processes, cellular components and molecular functions. The catalytic activity of molecular functions was the most enriched category with 73 (2.09%) genes, structural constituent of cytoskeleton with nine (0.25%) genes, and palmitoyl hydrolase activity with three (0.08%) genes. In biological processes, the metabolic process was enriched with 59 (1.69%) genes, and the microtubule category of the cellular process was enriched with nine (0.25%) genes, these were the most abundant down-regulated genes. 

**Figure 6:**
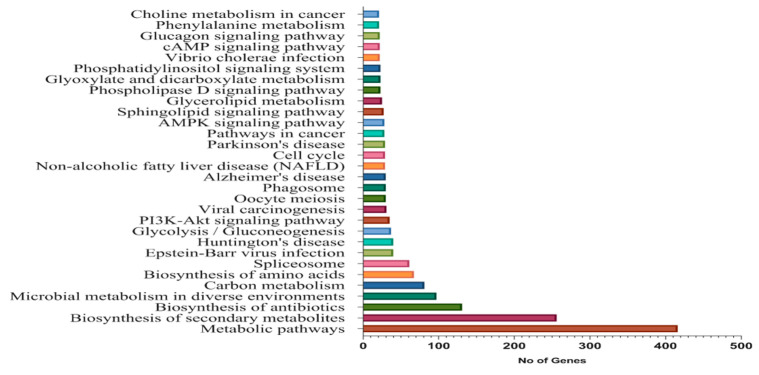
KEGG pathway enrichment analysis of DEGs.

A threshold of top 30 was set for KEGG pathways analysis (Figure 6). A total of 416 genes were enriched in metabolic pathways, followed by 256 genes in biosynthesis of secondary metabolites, and 131 genes involved in biosynthesis of antibiotics. These were the most abundant genes involved in KEGG pathways.

### 3.4. Gene Co-Expression Correlation Network Analysis 

**Figure 7:**
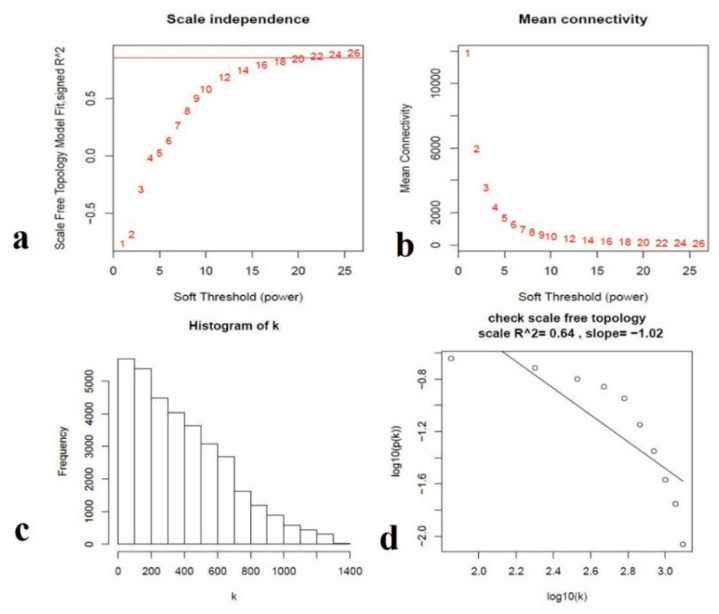
Determination of soft-thresholding power in the gene weighted co-expression network analysis. (**a**) Analysis of the scale-free fit index for various soft-thresholding powers (β). (**b**) Analysis of the mean connectivity for various soft-thresholding powers. (**c**) Histogram of connectivity distribution when β = 8. (**d**) Checking the scale-free topology when β = 8.

The weighted co-expression correlation network analysis (WGCNA) is a bioinformatics tool that discovers the target genes network level of distinct genes [28,32,33]. The analysis of the co-expression network includes 12 RNA sequencing samples (Figure S1). The power of β = 8 (scale-free R^2^ = 0.64) was selected as a soft threshold to ensure a scale-free network (Figure 7a–d). To explore further understanding of the relationship of gene expression with progressive delayed gland morphogenesis and to identify the genes associated with gland formation, we performed co-expression analysis for total genes expressed in all samples of RNA-seq data.

**Figure 8:**
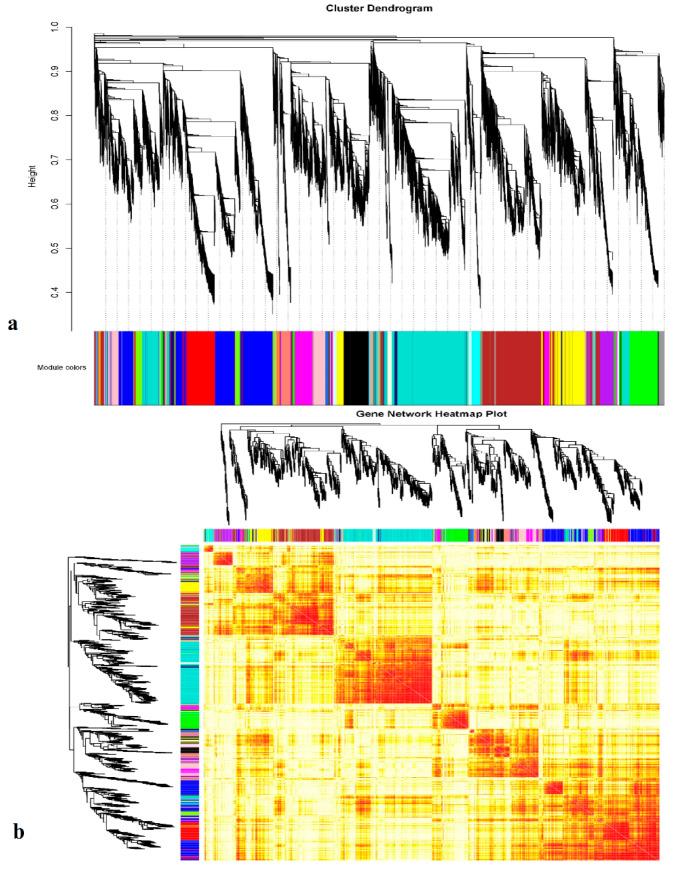
(**a**) Weighted gene co-expression network analysis (WGCNA), all expressed genes in Gbgl, Gbdd, Ga24h and Ga48h RNA-seq samples, showing hierarchical dendrogram co-expression of identified modules by WGCNA; each leaf represents a single gene. (**b**) The heat map plot of the gene network analysis. Dark red color shows overlap of highly expressed genes which pair from respective datasets, and the lighter color represents the low overlap genes. Different modules are indicated by the diagonals.

In the heat map plot for the gene expression network analysis, dark red color represents overlapped highly expressed module gene pairs from respective datasets, and yellow color indicates partially overlapped genes (Figure 8a). Furthermore, when analyzing the glandless imbibed seeds with glanded imbibed seeds and seedlings samples using the module trait relationship, 27 distinct modules were observed. From these 27 modules, only four modules were highly associated with glandless imbibed seed sample. Furthermore, in the description of 27 distinct modules, there were three modules that were highly correlated with glandless imbibed seed. The three modules, including MEbrown (r = 0.92, *p* = 2 × 10^−0^), MEmistyrose (r = 0.81, *p* = 0.001 × 10^−0^) and MEmagneta (r = 0.74, *p* = 0.006 × 10^−0^) were positively correlated with gland formation (Figure S2 and Table S2).

The MEbrown module contained 4552 genes that were related to genetic information of metabolic pathways. The module MEmistyrose consisted of 4910 genes, mainly involved in spliceosome and biosynthesis of secondary metabolites. The module MEmagneta contained 1543 genes, involved in biosynthesis of antibiotics (Table S3). For further study of the molecular mechanism of delayed gland morphogenesis, the unigenes of three modules were analyzed by gene ontology and KEGG pathways. In gene ontology analysis, unigenes were mostly related to protein binding (GO:005515), ATP binding (GO:0005524), the oxidation–reduction process (GO:0055114), protein phosphorylation (GO:0006468) and the membrane (GO:0016020).

### 3.5. Quantitative Real-Time PCR Validation of RNA-Seq Data

**Figure 9:**
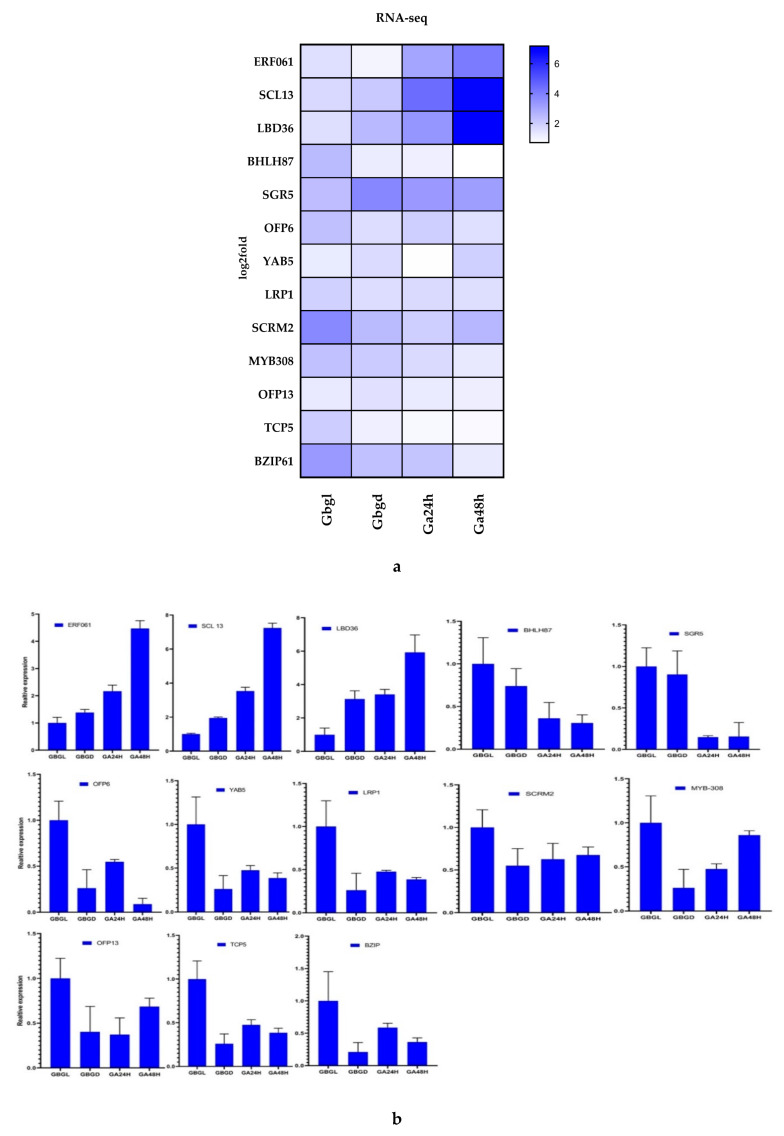
Validation of 13 DEGs related to delayed gland morphogenesis identified from transcriptome analysis data by quantitative reverse-transcription polymerase chain reaction (qRT-PCR). (**a**) RNA-seq-based log2-fold change expression; (**b**) qRT-PCR-based relative expression profile.

To confirm and validate the reliability of RNA-seq data, qRT-PCR analysis was performed to quantify the transcript level of selected genes. We selected 13 genes from DEGs (Table S4). Genes were involved in transcription factors (including *ERF061, BHLH87, OFP6, TCP5, SCRM2, BZIP61* and *OFP13*), protein synthesis (*SGR5, SCL13* and *LBD36*), axial regulatory (*YAB5* and *LRP1*) and MYB (*MYB308*). The transcript data from RNA-seq and qRT-PCR analysis were compared with the log2-fold change and relative expression level. qRT-PCR was performed by using three independent biological replicates. The primers are listed in Table S2. Housekeeping β-actin gene was used as a reference gene. The expression patterns of the 13 DEGs in the qRT-PCR results were highly consistent with transcriptome sequencing data, which further supported the reliability of our RNA-seq data. The genes *ERF061, SCL 13* and *LBD36* were up-regulated in RNA-seq data, while in qRT-PCR results, the relative expression pattern was higher than glandless imbibed seeds. The remaining genes *BHLH87, SGR5, OFP6, YAB5, LRP, SCRM2, MYB308, OFP13, TCP5* and *BZIP* were down-regulated in transcriptomic data and expressed lower than glandless imbibed seeds in qRT-PCR (Figure 9a,b). The results showed that RNA-seq data were reliable and conducive to the identification of DEGs in gland development on imbibed seeds and seedlings.

## 4. Discussion

Several genome sequencing projects of diploid cotton plants have been completed on the cotton genome [34,35,36]. In this experiment, we identified the genes which play a key role of pigments/glands underlying delayed gland morphogenesis. The cotton seed is a source of high quality protein (23%) and oil (21%) [22], although the utilization of nutrients resources is hampered due to presence of pigmented glands with gossypol toxicity [23]. Ever-increasing demand for food for human consumption has led scientists to explore the potential of cotton seed as a source of food and oil. RNA-seq was employed to discover the expression profile and sort out the candidate genes from DEGs related to delayed gland morphogenesis. RNA-seq is a useful tool to discover whole genome expression and screen out the candidate genes from DEGs [21]. In previous studies, RNA-seq studies were applied in different traits of fibers [20,37,38,39], with biotic and abiotic stresses to identify the related genes [40,41]. In this experiment, we compared DEGs of glandless imbibed seeds of *G. bickii* with glanded imbibed seeds of *G. arboreum*, and seedlings of both species. There is a delayed gland morphogenesis trait in Australian *Gossypium* species where pigment gland is formed after seed germination, and then the gossypol appears [42,43]. To gain accurate and repeatable RNA-seq data, we have used three biological replications from each sample, and the average number of clean reads (92.17%) of the Q30 and GC content percentage was about 43.47% and the average of each sample 92.93%. High correlation value was observed between the replicates of *G. bickii* seedling (0.97), which indicates the reliability and quality of RNA-seq data and sampling. In this study, we compared the wild Australian species *G. bickii* glandless imbibed seeds with glanded seedling and *G. arboreum* glanded imbibed seeds with seedling.

### 4.1. Comparison of Expression Profiles, RNA Sequences between Glandless G. bickii and Glanded G. arboreum 

At imbibed seed and seedling stages, a comparison of differentially-expressed genes showed that a large number of genes were significantly expressed in Gbdd vs. Ga48h at the seedling stage, while very few DEGs were differentially expressed in Gbgl vs. Ga24h at the imbibed seed stage, which suggested a highly diverged gene expression pattern. The transcription factors gene was reported to regulate many pathways during plant development and gland formation in the cotton plant [22]. Some MYC gene was reported as an important regulator of trichrome and pigmentation on the leaf of *Arabidopsis thaliana* [44,45]. In our study, we also found some transcription factors, protein synthesis, axial regulatory and MYB genes related to delayed gland morphogenesis. These genes were selected based on expression and pathway analysis. Previous studies identified that several WRKY transcription factor gene families were reported with 74 subfamilies identified in *A. thaliana,* which participates in regulation of various developmental processes of pigmentation [46]. The differentially-expressed genes (*ERF61, BHLH87, OFP6, TCP5, SCRM2, BZIP61* and *OFP13*) were found to be involved in transcription factor activities. While remaining DEGs were related to protein synthesis, axial regulatory and MYB-related function of these genes on leaf trichrome and pigmentation on *A. thaliana*, and we confirmed their expression through qRT-PCR. Validation and functional annotation of these genes can provide better insights into the mechanism underlying the genes by CRISPR-cas9, virus-induced genes silencing and overexpression molecular techniques.

### 4.2. Hub Genes Identified Using WGCNA 

In the present study, we used WGCNA to identify the modules associated with delayed gland morphogenesis. Here we used weighted gene co-expression network analysis to identify the significant modules of specific genes associated with delayed gland morphogenesis, and identified several modules highly associated with glandless imbibed seed. There were four modules highly associated with glandless imbibed seed samples. These module genes involved in transcription factors play a key role in growth, development and gland formation in cotton [47]. Therefore, these hub genes are also worthy of further research.

## 5. Conclusions

To understand the differences in gene expression pattern between two species, *G. arboreum* and *G. bickii*, the genes related to glandless trait on seeds were identified. Here we performed RNA-seq and constructed 12 libraries from four different samples, including two from *G. arboreum* imbibed seed and germination stage and two from *G. bickii* (glandless imbibed seed and glanded seedling). DEGs were identified using Blast2GO software and we identified genes related to gland formation in cotton. Gene ontology analysis identified and categorized different genes based on their function and up-regulation and down-regulation expression profiles. KEGG pathways analysis revealed that the genes showed a contrasting expression trend in different pathways, i.e., metabolic pathway, biosynthesis of secondary metabolites and biosynthesis of antibiotics, and the genes controlling these pathways were involved in different transcription factors.

Co-expression network analysis has identified four modules that were highly associated with delayed gland morphogenesis. These findings have revealed promising candidate genes for improvement of glandless imbibed seed and plant in further molecular studies of upland cotton.

## Figures and Tables

**Table 1:** Summary of 12 separately pooled RNA sequencing read counts, using *Gossypium arboreum* genome as a reference genome.

Sample Name	Raw Reads	Clean Reads	Clean Bases	Error Rate (%)	Q20 (%)	Q30 (%)	GC Content (%)
Gbdd_1	76,722,280	75,666,498	11.35G	0.03	97.53	93.05	43.94
Gbdd_2	68,409,156	67,453,570	10.12G	0.03	97.63	93.31	43.63
Gbdd_3	75,015,858	74,123,028	11.12G	0.03	97.65	93.34	43.57
Ga48h_1	70,605,862	69,735,728	10.46G	0.03	97.45	92.81	43.91
Ga48h_2	77,498,638	76,604,382	11.49G	0.03	97.57	93.13	43.95
Ga48h_3	76,083,930	75,133,632	11.27G	0.03	97.37	92.64	43.77
Gbgl_1	77,882,552	77,011,776	11.55G	0.03	97.32	92.62	43.24
Gbgl_2	66,734,644	65,760,564	9.86G	0.03	97.2	92.36	43.25
Gbgl_3	70,721,200	69,789,014	10.47G	0.03	97.61	93.24	43.67
Ga24h_1	70,796,734	69,833,972	10.48G	0.03	97.62	93.21	43.04
Ga24h_2	82,478,328	80,547,802	12.08G	0.03	97.65	93.32	43.67
Ga24h_3	74,698,432	73,859,056	11.08G	0.03	97.17	92.17	43.25

Gbdd, Ga24h, Gbgl and Ga48h represent *Gossypium bickii* glanded seedlings, *Gossypium arboreum* glanded imbibed seeds, *G. bickii* glandless imbibed seeds and *G. arboreum* glanded seedlings, respectively.

## Supplementary Materials

The following are available online at https://www.mdpi.com/2073-4425/11/5/472/s1: Figure S1. 12 RNA sequencing samples were included in co-expression network analysis, clustering dendrogram of 12 RNA sequence samples. Figure S2. Number of modules correlated with glandless trait. Table S1. List of differentially-expressed genes. Table S2. List of module size consisting of number of genes in each module. Table S3. Number of genes identified in modules. Table S4. List of primers used for qRT-PCR validation.

## Abbreviations

Gbgl	*Gossypium bickii* glandless imbibed seed
Gbdd	*Gossypium bickii* glanded seedlings
Ga24h	*Gossypium arboreum* glanded imbibed seed
Ga48h	*Gossypium arboreum* glanded seedlings
ME	Module Eigengenes
